# Detection of H-type bronchoesophageal fistula in a newborn: A case report and literature review

**DOI:** 10.1097/MD.0000000000025251

**Published:** 2022-02-25

**Authors:** Huaying Li, Li Yan, Rong Ju, Biao Li

**Affiliations:** a Department of Neonatology, Chengdu Women's and Children's Central Hospital, School of Medicine, University of Electronic Science and Technology of China, Chengdu, China; b Department of Respiration Center, Children's Hospital of Chongqing Medical University, Ministry of Education Key Laboratory of Child Development and Disorders, Chongqing, People's Republic of China.

**Keywords:** bronchoesophageal fistula, bronchoscopy, esophagoscopy, newborn

## Abstract

**Rationale:**

Congenital tracheoesophageal fistula (TEF) is a rare developmental malformation. The H subtype accounts for approximately 4% of TEFs. Unlike other TEFs, the H-type is not accompanied by esophageal atresia and has nonspecific clinical symptoms, and its specific anatomical abnormalities are not always readily apparent. Furthermore, none of the currently available diagnostic methods for H-type TEF have absolute sensitivity, resulting in misdiagnoses, and accurate diagnoses are often delayed even until adulthood; in our case, we detected a congenital bronchoesophageal fistula, which is even more rare than regular H-type TEF, through a technique that was not previously reported for newborns, involving bronchoscopy, with methylene blue injected through an esophagoscope. We believe that we have provided this kind of case first in newborns.

Furthermore, because there is not one literature summarizing the clinical symptoms and the effective methods up to now, we still are not clear which detective method is more efficient or accurate, especially in newborns, so it is very necessary to summarize and compare for improving the early diagnosis of TEFs; our study makes a significant contribution to the literature because we collated previously reported cases, including the clinical features and the usefulness and success rates of major tests, which will be very helpful for the early diagnosis of TEFs.

**Patient concerns:**

A newborn male presented with an array of nonspecific clinical symptoms from birth, leading to pneumonia and mechanical ventilation. Oral feeding led to an improvement in most but not all symptoms, which returned when oral feeding was resumed. A second round of confirmatory tests was still unable to detect the cause.

**Diagnosis:**

The diagnosis of H-type bronchoesophageal fistula was established through a technique that was not previously reported for newborns, involving bronchoscopy, with methylene blue injected through an esophagoscope.

**Interventions:**

The surgery was performed after diagnosis, and the bronchoesophageal fistula was successfully repaired.

**Outcomes:**

The patient was discharged on postoperative day 7, and his status was reported to be normal at a follow-up visit 8 months after surgery.

**Lessons:**

H-type TEF is a rare congenital abnormality, and its early diagnosis is highly difficult, especially bronchoesophageal fistula. Increased oral saliva and air-filled stomachs are characteristic manifestations. Bronchoscopy combined with esophagoscopy can improve the rate of early diagnosis. A combination of tests can improve the detection rate.

## 1 Introduction

Congenital tracheoesophageal fistulas (TEFs) are rare, occurring in approximately 1 in 2500 live births.^[1]^ TEFs have been classified into 5 types: types I to IV are commonly associated with esophageal atresia and are readily diagnosed and treated after birth. Type V (“H-type”) is the most rare, consisting of an isolated TEF without esophageal atresia, constituting about 4% of all congenital TEFs.^[2]^ Due to its nonspecific symptoms, the small size of the fistula in infancy, H-type TEF is rarely diagnosed in the neonatal period. As a result, patients endure constant aspiration and recurrent pulmonary infections, tracheomalacia, and bronchiectasis; death in infancy also occurs. Infants who present with aspiration, coughing, and choking during meals are commonly misdiagnosed as having reflux.^[3]^ Accurate diagnoses are difficult even with the use of a barium swallow, computed tomography (CT) scan, isolated bronchoscopy, or esophagoscopy, and the condition can remain undiagnosed until adulthood.^[4,5]^ No commonly used tests have 100% sensitivity.^[6]^ Furthermore, patients with severe conditions cannot be separated from mechanical ventilation, increasing the difficulty of diagnosis. Thus, it is problematic to detect H-type TEF using a single diagnostic approach, especially bronchoesophageal fistula. This report describes a case of a newborn with H-type bronchoesophageal fistula, presents a novel clinical approach for identification in the neonatal period and reviews the use and success rates of current diagnostic methods.

## 2 Ethics statements

Informed written consent was obtained from the parents after the nature of the study had been fully explained to them. The parents of the patients provided informed consent for publication of the case.

## 3 Case presentation

**Figure 1:**
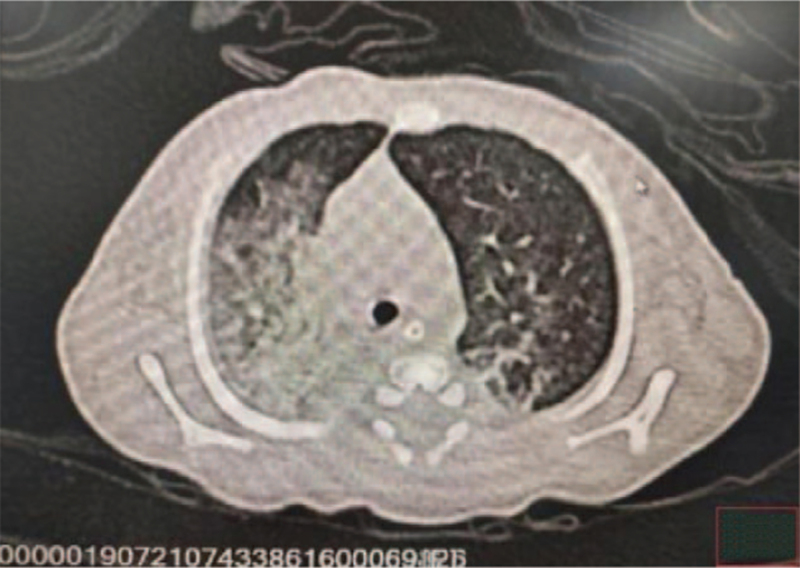
The CT showed no abnormality about bronchus and esophagus.

**Figure 2:**
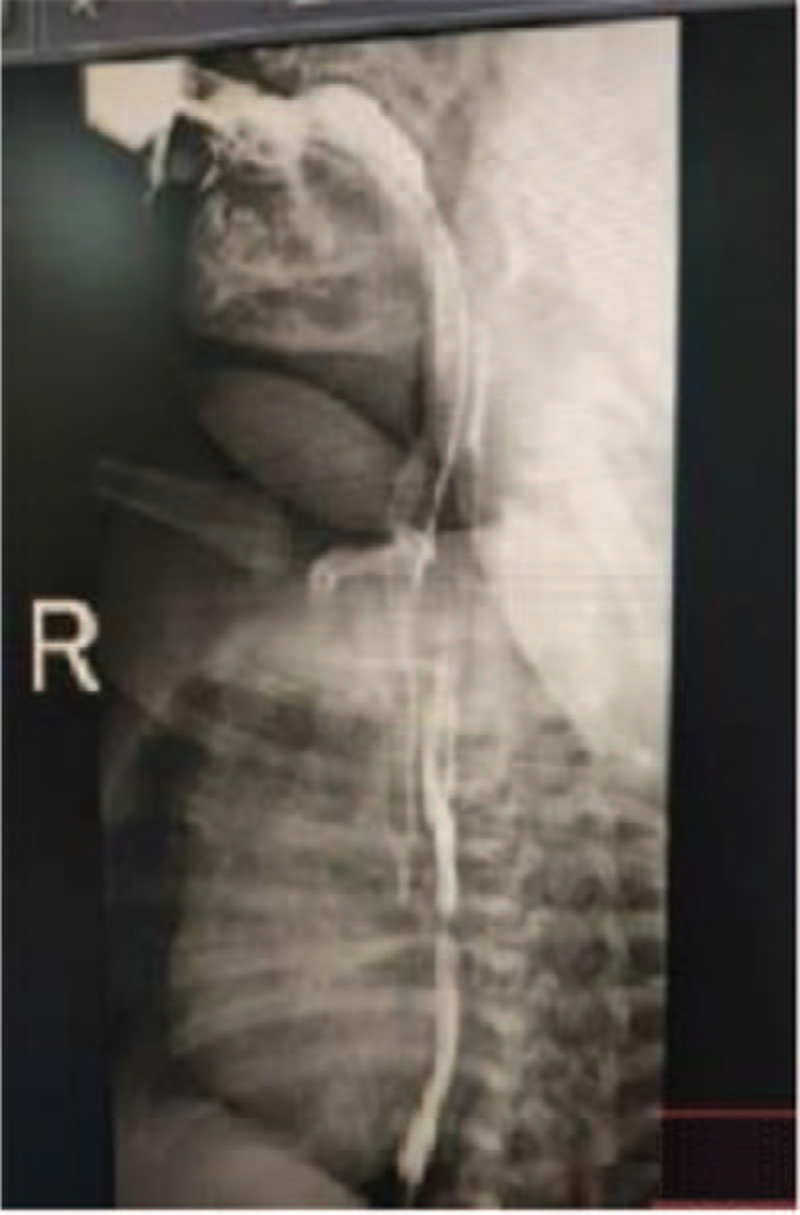
The barium esophagoram did not demonstrate the fistulas.

**Figure 3:**
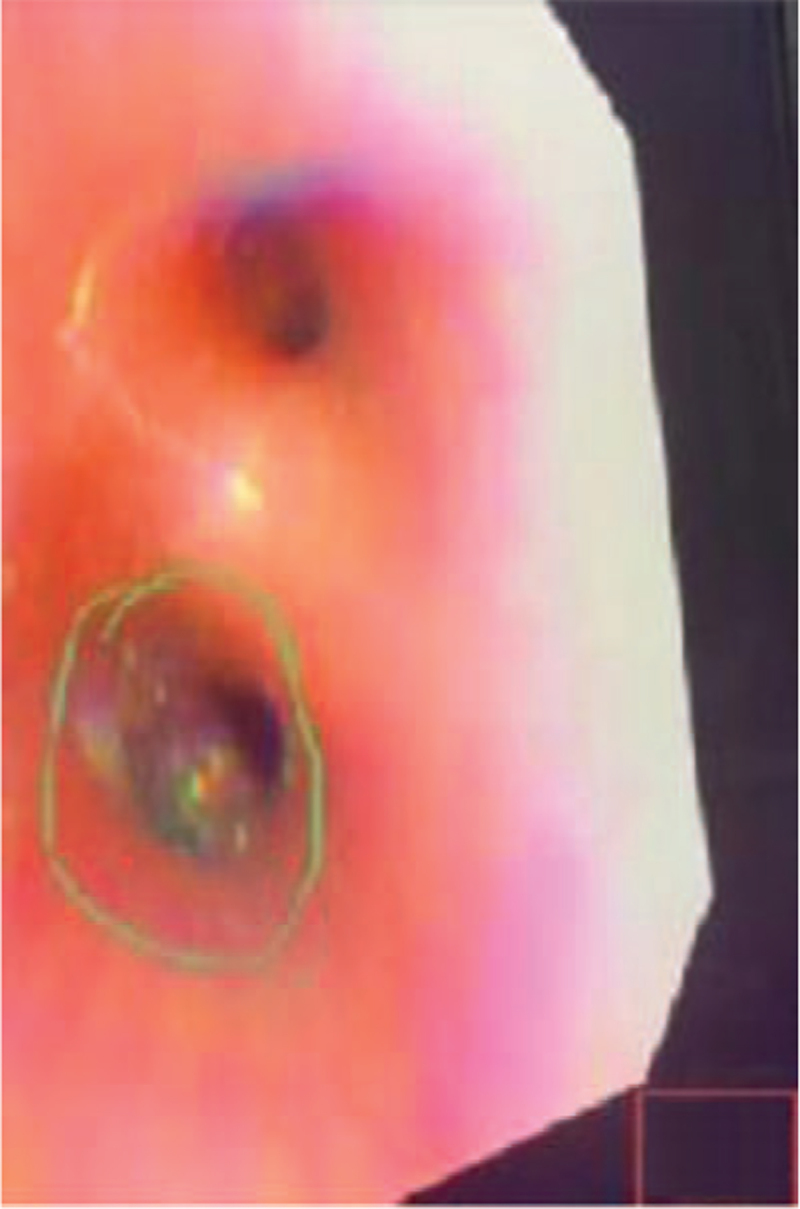
Blisters are suggested in the lower left bronchus (circled area), but are not definitive because of the sputum levels.

A 35-week gestation male newborn presented postnatally with excessive oral secretions, noisy breathing accompanied by mucus, cyanosis, and apnea. Her antenatal history and physical examination results were normal. Chest radiography revealed mild inflammation. He was able to consume food normally. On the second day, his breath became difficult, and he required endotracheal intubation and ventilator support. Chest radiography revealed right lower lobe pneumonia, while cardiac ultrasound showed a patent foramen ovale, and cranial and gastroesophageal reflux ultrasounds were normal. His hemoglobin level was 135 g/L. These results suggest a resolution of pulmonary infection. However, we found that his stomach was dilated and filled with air, although he underwent mechanical ventilation with continuous gastrointestinal decompression. We suspected that the TEF released air into the stomach. We performed a barium swallow, chest CT, and isolated bronchoscopy in sequence, but did not detect any anomalies (Figs. 1 and 2). Several days after stopping oral feeding, the chest X-ray showed significant improvement, and the child was weaned from the mechanical ventilator and resumed oral feeding. However, only 2 days later, pulmonary inflammation was again detected, and mechanical ventilation was re-implemented. Infection markers were negative. Chest radiography showed severe inflammation in the right and left lobes. We again performed a three-dimensional CT airway reconstruction and barium swallow, and performed a repeat bronchoscopy. There was an indication of blisters in the lower left bronchus, but a definitive assessment was not possible because of the sputum levels (Fig. 3).

**Figure 4:**
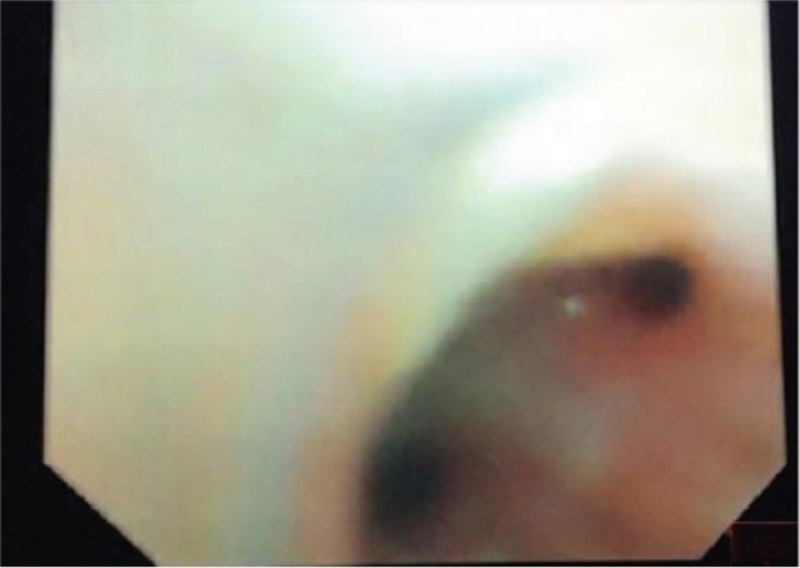
Methylene Blue can be seen in the left lung bronchus via bronchoscope.

Our team hypothesized that excessive oral secretions, recurrent pulmonary infection, and continual stomach expansion during mechanical ventilation most likely resulted from communication between the trachea and esophagus. Thus, we conducted a bronchoscopy with methylene blue (MB) injected through an esophagoscope. As anticipated, the MB was seen on bronchoscopy in the left lung bronchus (Fig. 4), revealing an H-type bronchoesophageal fistula between the esophagus and the bronchus.

TEF was confirmed during surgery and was successfully repaired. The patient's symptoms disappeared, and he was weaned from the ventilator. Three days later, a chest radiograph demonstrated marked improvement in the pulmonary inflammation. In the following days, symptoms did not recur when the diet was gradually advanced to full oral feeding. The infant was discharged soon after the operation, and his status was reported to be normal at a follow-up visit 8 months after surgery.

Informed consent was obtained from the patient's guardian for the purpose of publication.

## 4 Discussion

Congenital H-type TEF is difficult to detect because of its anatomical features.^[5]^ The low morbidity and high misdiagnosis rate lead to few reports in the neonatal period, and as an unknown number of cases die before diagnosis, the actual incidence is likely higher than reported. Most cases presented with symptoms during the neonatal period^[2,3,7–11]^ are rarely diagnosed within the first month and many are not diagnosed until adulthood. In the interim, they suffer from recurrent pulmonary infections and are often repeatedly hospitalized. The development of severe depression has also been reported.^[5,12]^ Most patients underwent multiple tests, including chest computed tomography (CT) scans, barium swallows, isolated bronchoscopies, and esophagogastroduodenoscopy (EGD) with few TEFs detected. Suen (2018) reported on 3 adults who were eventually diagnosed with H-TEF and received fundoplication for gastroesophageal reflux disease (GERD), gastrostomy, and/or cardiac catheterization.^[3,5]^

Early identification and treatment of H-TEF are important, and the critical question is, “Why is it so often missed?” An important consideration is that most early clinical symptoms are nonspecific.^[13]^ Early clinical symptoms reported in the literature include coughing with every feed, choking, emesis, cyanosis, stridor with feeding, hemoptysis, regurgitation, shortness of breath, tachypnea, and recurrent pneumonia, and rarely, “noisy breathing with excessive mucus, abdominal distension, and drowning from excessive saliva”. Among these symptoms, coughing, cyanosis, choking, and recurrent pneumonia were the most common (Table 1).^[5]^ In our case, the prominent clinical manifestations were increased oral secretion, air-filled stomach, and paroxysmal cyanosis without obvious cough. Of these, increased oral secretion and air-filled stomachs were the key clues. Although the vast majority of older patients develop cough due to H-TEF, in the early neonatal period, the cough mechanism is not yet fully developed, and some newborns require immediate mechanical ventilation, confounding the predictive value of the symptoms. Therefore, the actual presence of coughing should not be a diagnostic clue for an H-type TEF.

**Table 1:** Literature review.

Author	Year	Age of onset	Age of diagnosis	Symptoms	Inspection methods	Times of check	Confirm methods
Peter Mattei^[2]^ (1 case)	2012	1 d	Infant	Coughing with every feed	Chest radiograph contrast esophagram bronchoscopy	2	Bronchoscopy
Timothy E.S Mith^[3]^ (1 case)	2010	Birth	7 m	Copious oral secretions aspiration pneumonitis Choking and cough	Upper gastrointestinal radiologic studies Brochoscopy radiological pharyngeal function study Barium esophagram (confirm)	4	Barium esophagram
Ahmed H^[4]^ (23 cases)	2016	/	5 d–3 y	Chocking and coughing during feeds (52.2%) recurrent chest infection (69.6%) cyanosis (43.5%). One presented with abdominal distension	Bronchoscopyc Esophagogram		Esophagogram In 11 (47.8%), a single study confirmed the diagnosis, 8 (34.8%) required two studies while 4 (17.4%) required three studies
Hon Chi Suen^[5]^ (3 cases)	2018	Baby	32 y	Throwing up coughing and regurgitation recurrent pulmonary infection severe depression	Barium swallow EGD CT scan esophagoscopy	4	CT scan
		Baby	49 y	Frequent respiratory infection shortness of breath	Barium swallow	1	Barium swallow
		/	55 y	coughing during swallowing be drown by his own saliva	CT scan Sagittal reconstruction of the CT	2	Sagittal reconstruction of the CT
N. González Temprano^[6]^ (2 cases)	2014		2+m	Pneumonia Pneumonia atelectasis	esophagogram	1	Esophagogram
		/	1+m	Hypoxemia apnea	Bronchoscopy	1	Bronchoscopy
Victoria K. Pepper^[7]^ (1 case)	2017	1 d	33 d	Aspiration pneumonitis or respiratory distress syndrome of prematurity	Bronchoscopy	1	Bronchoscopy
Dong Yun Lee^[8]^ (1 case)	2012	From birth	3 m	Choking and recurrent aspiration	Radionuclide salivagram	1	Radionuclide salivagram
Nitin Pant ^[9]^ (1 case)	2013	Birth	1+m	Cough fever, hiccup regurgitation after feeds pneumonias.	A non-ionic water Soluble contrast esophagogram, tracheobronchoscopy	1	Tracheobronchoscopy
Anko Antabak^[10]^ (1 case)	2015	Birth	/	cyanosis, dyspnea and tachypnea.	Gastrografin esophogram	1	Gastrografin esophogram
Fallon SC^[11]^ (102 cases)	2017	Birth	2–1427 d	Cyanosis, choking with feeding	Fluoroscopy Endoscopy	1–2	Fluoroscopy Endoscopy
Dionisios^[12]^ (1 case)	2011	/	57 y	Fever, fatigue, and weight loss	CT esophagoscopy	2	Esophagoscopy
I-Chen Chen^[13]^ (1 case)	2014	Birth	21 d	tachypnea Recurrent pneumonia	Esophagogram\MDCT (failure) Tracheobronchoscopy (suspecte)	3	Tracheobronchoscopy
Deng, Z. H^[14]^ (2 cases)	2012	From birth	5 y 4 y	drown by his own saliva Choking Tachypnea Recurrent pneumonia Intermittent abdominal distention	bronchoscopy combined with esophagoscopy	2	Bronchoscopy combined with esophagoscopy
Jiangtao Dai ^[15]^ (31 cases)	2018	/	/	choking while consuming milk, coughing, and shortness of breath	Iodine oil contrast bronchoscopy combined with esophagoscopy three-dimen sional computed tomography (3-D CT) reconstruction	1–3	Iodine oil contrast(16) bronchoscopy combined with esophagoscopy (11) 3-D CT(4)
NG^[16]^ (5 cases)	2006	Birth	7 d 9 d 16d 14d 58 d	desaturation after feeds and/or aspiration pneumonia	contrast study/tube esophagogram (all cases); chest radiograph(4 cases); bronchoscopy (3 cases); esophagoscopy.(1cases);		Esophagogram (most sensitive, identify in all 5 cases)
By Albert Ko^[17]^ (1 case)	2000	/	15 m	Coughing, choking, emesis, pneumonias	Esophagogram	1	Esophagogram
D.V.Le^[18]^ (1 case)	2001	Birth	5 m	Poor feeding Repeated vomiting	three-dimensional computed tomography (3-D CT) Bronchoscopy esophagoscopy	3	Esophagoscopy
Conrad R^[19]^ (4 case)	1979	/ / 5w 40y	1y 6 y 12 y 50 y	Cough with choking(1 y) Noisy breathing with much mucus (6 y) Chronic aspiration(12y) Hemoptysis, severe coughing	1y-radiologic studies 6 y-A barium swallow 12y-cine esophagogram 50 y-cine esophagogram	1 2 1 1	1.Esophagogram 2.cine esophagogram 3.cine esophagogram 4.cine esophagogram
A. Tarcan^[20]^ (1 case)	2003	Birth	44 d	Choking, cyanosis, coughing	Barium contrast rapid-sequence cine-esophagogram	1	Barium contrast
A Gunlemez^[21]^ (1 case)	2009	3 d	17 d	Apnea and aspiration pneumonia gastric dilatation	Bronchoscopy (failured) barium contrast rapid sequence cine-esophagogram (failured) MRI (succeed)	3	MRI
Shu Yi Sonia Lee,^[22]^ (1 case)	2018	Birth	15 m	recurrent respiratory infections coughing on feeding of fluids chronic microaspiration	fluoroscopy swallowing(suspection) Bronchoscopy and esophageogas troduodenoscopy	1	Bronchoscopy and esophageogastroduodenoscopy
Peter Donnelly,^[23]^ (1 case)	2016	Birth	23 d	Cyanotic, airway secretions a large leak from around the ETT	bronchoscopy	1	Bronchoscopy
Phalla Ou, MD^[24]^ (1 case)	2007	1 m	1 m	respiratory distress recur-rence of pneumonia	CT scane	1	CT scane
Muhammad Riazulhaq^[25]^ (1 case)	2012	Within 24 h	Within 24 h	Choking and cyanosis	Tube esophagram	1	Tube esophagram
Nilda M. Garcia, ^[26]^ (4 cases)	1998	/	15 d–3 m	Coughing, cyanosis, stridor with feeding	Bronchoscopy	1	Bronchoscopy
Josephiney Y^[27]^ (5 cases)	1997	/	/	GER recurrent respiratory tract infention tracheomalacia	CT scopy		CT scopy

/:no mention. Baby (under the age of onset): In some reports, the author only expressed in less than a year before the onset of disease. Times of check: How many checks were done before the diagnosis was confirmed. Inspection methods: All tests performed by the patients (not necessarily definitive). Confirm methods: The way the patient was finally diagnosed.

In previous reports, the most commonly used methods to detect H-type TEFs have been the barium swallow test,^[13]^ followed by bronchoscopy and chest CT scans. The least common methods are three-dimensional airway reconstruction, esophagoscopy, salivagram, and MRI. Only 2 studies have reported cases of bronchoscopy tracer via esophagus MB injection in older infants or adults ^[14,15]^ (Table 1). We are unaware of previous use of this method in newborns.

None of the tests that are currently in use for diagnosing H-type TEF showed 100% sensitivity. In a multicenter study, fewer than half of the H-type TEF cases were confirmed by a single contrast study.^[4]^ Based on previously reported data, the success rates of major tests, from high to low, are upper digestive tract contrast studies (including contrast esophagram, barium esophagram, iodine oil contrast, gastrografin esophagram, cine esophagram, fluoroscopy swallowing, tube esophagram), bronchoscopy, chest CT, and esophagoscopy. The lower usage of other types of tests makes it difficult to evaluate their success rates. For example, bronchoscopy tracer via esophageal MB injection, three-dimensional airway reconstruction of the CT, MRI, and salivagram (Table 1).

These calculations show that H-type TEFs should be detectable through the combined use of upper digestive tract contrast studies, bronchoscopy, and chest CT scans. Therefore, the question remains: “Why is it still often missed?”

H-type TEFs are always more cephalad on the tracheal side and lower on the esophagus side, making the anatomical construction more like N than H.^[5]^ As a result, a horizontal axial view of the chest CT did not show the entire fistula. Otherwise, if the fistula is thin, the opening is small and lower on the esophageal side, and the barium may not pass through it. A fistula can also be missed during bronchoscopy if it is closed in the inhalation phase or connected to the bronchus rather than the trachea, or if there is excess phlegm, or if the patient cannot be separated from a ventilator for the examination. Use of an esophagoscopy tracer via tracheal MB injection also carries the risk of patient choking and asphyxia. In our case, H-type bronchoesophageal fistula was more difficult to detect through bronchoscopy because of the newborn's narrow airway, and after many failures with other tests, our use of a bronchoscopy tracer via esophageal MB injection was successful in detecting the fistula. MB is a heteroaromatic compound used as a staining material or medicine, is commonly used to detect fistulas, passes readily through even small fistulas, and is easily observed by bronchoscopy. Our report is the first to document this successful method in newborns. The process was accomplished efficiently, no complications occurred, and the patient recovered without any adverse reactions. The widespread use of our method will enable earlier diagnosis of patients with H-type TEF, especially broncho-bronchoesophageal fistula, ending their suffering earlier.

Our use of bronchoscopy combined with esophagoscopy is a novel approach to the diagnosis of H-type tracheoesophageal fistula in a newborn, especially bronchoesophageal fistula. H-type TEFs affect patients’ quality of life from infancy to adulthood, and effective and sensitive diagnostic measures are needed. However, their low incidence, nonspecific clinical features, and anatomical structure lead to misdiagnosis and delayed diagnosis. Until improved measures become available, we noted the diagnostic value of specific manifestations (increased oral saliva, air-filled stomach) and an effective method of confirmation (bronchoscopy tracer via esophageal MB injection). The use of these methods to detect H-type TEFs in the newborn period will improve the rate of early diagnosis, reduce medical expenses, and avoid prolonged suffering.

Bronchoesophageal fistula is very rarely. We believe that we provide this kind of case first in newborns and our study maked a significant contribution to the literature because we collated the previously reported cases including the clinical featues and the usage and success rates of major tests, that will be very helpful to the early diagnosis of TEFs.

## Author contributions

**Conceptualization:** Huaying Li.

**Data curation:** Huaying Li, Rong Ju, Li Yan, Biao Li.

**Formal analysis:** Huaying Li, Rong Ju, Li Yan.

**Funding acquisition:** Huaying Li.

**Investigation:** Huaying Li, Rong Ju, Biao Li.

**Methodology:** Huaying Li, Rong Ju, Li Yan.

**Project administration:** Huaying Li, Rong Ju.

**Resources:** Huaying Li, Rong Ju, Li Yan, Biao Li.

**Software:** Huaying Li, Biao Li.

**Supervision:** Huaying Li, Rong Ju.

**Validation:** Huaying Li, Rong Ju.

**Visualization:** Huaying Li, Rong Ju.

**Writing – original draft:** Huaying Li, Li Yan, Biao Li.

**Writing – review & editing:** Huaying Li, Rong Ju.
